# Supplementary material for: Methods for the Inclusion of Real-World Evidence in a Rare Events Meta-Analysis of Randomized Controlled Trials
Source: J Clin Med. 2023 Feb 20;12(4):1690. doi: 10.3390/jcm12041690 (PMC9964527; doi:10.3390/jcm12041690)
Supplement: Supplementary file 1 [file jcm-12-01690-s001.zip › jcm-2095717-supplementary.pdf]

R code for the methods included this study

Please install the following packages first

install.packages(c("rstan"))

y and y_se represent the vector of effect and standard error, respectively. J denotes the number of studies included in a meta-analysis.

The naïve data synthesis

```
data {
   int<lower=1> J;
   real y[J];
   real<lower=0> y_se[J];
}

parameters {
     real theta;                    // relative treatment effect (log OR)
     real<lower=0> tau;        // the heterogeneity parameter
   }

model
  {
         //prior distributions
         tau ~ normal (0, 0.5);
         theta ~ normal (0,2.82);
         //likelihood
         For (i in 1:J)
           {
           or1[i]~normal (theta, y_se[i]+tau);}
   }
```

The design-adjusted synthesis

```
data {
   int<lower=1> J;
   real y[J];
   int<lower=0, upper=1> group[J];      // the study type variable, where 0 represents the RCTs and
                                                    1 represents the RWE studies.
   real<lower=0> y_se[J];
   int<lower=1> R;      // the number of RWE studies
}
```

```
parameters {
     real<lower=0,upper=1> w[R];      //the variance inflation factor
     real theta;
     real<lower=0> tau;
   }

model {
   //prior distributions
     theta~ normal(0, 2.82);
     tau ~ normal(0, 0.5);
     w ~ beta(0.25,1);       // w ~ beta(0.25,1), w ~ beta(1.5,1), w ~ beta(4,1) indicate that we placed a low, medium, and higher level confidence in RWE studies, respectively.
     //likelihood
       for( i in 1:J)
          {if (group[i]==0) or1[i]~normal(theta,orse[i]+tau ); //0 denote RCT
           else      or1[i]~normal(theta,orse[i]/q[i-R]+tau); }
}
```

The using real-world evidence as prior information

```
data {
   int<lower=1> M;      // the number of RCTs
   real y_rct [M];      // the effect in RCTs
   real<lower=0> y_se_rct[M];      //the standard error in RCTs
   real mu;      //the estimated effect of the RWE studies
   real se_rwe;      //the standard error of the estimated effect of the RWE studies
}

parameters {
     real theta;
     real<lower=0> tau;
     real<lower=0,upper=1> w;      // the variance inflation factor
}

transformed parameters {
  real s= se_rwe/sqrt(w);
}

model {
   //prior distributions
     theta~ normal(mu, s);
     tau ~ normal(0, 0.5);
     w ~ beta(0.25,1); // w ~ beta(0.25,1), w ~ beta(1.5,1), w ~ beta(4,1) indicate that we placed a low, medium, and higher level confidence in RWE studies, respectively.
     //likelihood
```

```
        for( i in 1:M)
            { y_rct[i]~normal(theta, y_se_rct[i]+tau); }}
```

The three-level hierarchical models

```
data {
    int<lower=1> J;
    int<lower=0, upper=1> group[J];
    vector[J] y;
    vector[J] y_se;
}
parameters {
    vector [2] theta_design;
    real theta;
    real<lower=0> tau;
    real<lower=0> tau_rct;
    real<lower=0> tau_rwe;
  real<lower=0,upper=1> w;
}
model {
      //prior distributions
      vector[J] y_hat;
      theta ~ normal(0, 2.82);
      tau ~ normal(0, 0.5);
      tau_rct ~ normal(0, 0.5);
      tau_rwe ~ normal(0, 0.5);
      for (i in 1:J)    y_hat[i] = theta_design[group[i]];
      theta_design ~ normal (theta, tau);
      w ~ beta(0.25,1); // w ~ beta(0.25,1), w ~ beta(1.5,1), w ~ beta(4,1) indicate that we placed a
low, medium, and higher level confidence in RWE studies, respectively.
//likelihood
      for (i in 1:J){
      if (group[i]==0) {y[i] ~ normal(y_hat[i], tau_rct+y_se[j]);} else {
        y[i] ~ normal(y_hat[i], tau_rwe /w+y_se[i]);}
    }
}
```
